# Comparative Analysis of Interleukin-6 Levels in Polycystic Ovary Syndrome (PCOS) Patients With and Without Insulin Resistance

**DOI:** 10.7759/cureus.81878

**Published:** 2025-04-08

**Authors:** Shah Zeb, Reema Gul, Ashraf, Rumman

**Affiliations:** 1 Internal Medicine, Bacha Khan Medical College, MTI Mardan Medical Complex, Mardan, PAK; 2 Gynecology, MTI Mardan Medical Complex, Mardan, PAK; 3 Research and Development, Pro-Gene Diagnostics and Research Laboratory, Mardan, PAK; 4 Pulmonology, MTI Mardan Medical Complex, Mardan, PAK; 5 Pharmacovigilance/Active Drug Safety Monitoring and Management, Association for Community Development, Peshawar, PAK

**Keywords:** hormonal imbalances, inflammatory markers, insulin resistance, interleukin-6 (il-6) levels, polycystic ovary syndrome (pcos)

## Abstract

Background

Polycystic ovary syndrome (PCOS) is an endocrine disorder prevalent in reproductive-aged women, characterized by hyperandrogenism, ovulatory dysfunction, and polycystic ovaries. It is often associated with insulin resistance, increasing risks for metabolic disorders such as diabetes and cardiovascular disease. Elevated interleukin-6 (IL-6) levels indicate chronic inflammation, a key component in PCOS pathogenesis, though its precise role and diagnostic potential remain unclear.

Objective

This study aims to evaluate the relationship between IL-6 concentrations and the severity of insulin resistance in patients with PCOS and to assess the potential of IL-6 as a diagnostic biomarker for insulin resistance within these patients.

Methodology

This cross-sectional study was carried out from February 2024 to January 2025 at the Departments of Medicine and Gynecology, Mardan Medical Complex Teaching Hospital, Khyber Pakhtunkhwa, Pakistan. Patients were divided into insulin-resistant and non-insulin-resistant groups based on clinical assessments. Pearson correlation and linear regression analyses were employed to investigate the relationship between IL-6 levels and study variables, adjusting for significant correlations and potential confounders. The relationship between insulin resistance and IL-6 levels was examined through linear regression. The study also evaluated IL-6's predictive ability for insulin resistance using receiver operating characteristic (ROC) curve analysis, calculating the area under the curve (AUC), with a significance threshold set at p<0.05.

Results

This study includes 112 patients with PCOS, and significant differences were identified between insulin-resistant (39.29%, 44 patients) and non-insulin-resistant patients (60.71%, 68 patients). IL-6 levels were notably higher in insulin-resistant patients (287.00±84.33 pg/mL) compared to non-resistant patients (159.16±52.36 pg/mL). PCOS patients revealed a complex relationship better described by a simple linear regression model (R²=0.56). Additionally, ROC curve analysis demonstrated the classifier's high diagnostic accuracy (AUC=0.91)

Conclusion

IL-6 levels in PCOS patients were significantly influenced by a combination of physiological, metabolic, and symptomatic factors. BMI, polycystic ovaries, and specific metabolic markers emerged as key predictors, underscoring the multifaceted role of IL-6 in the pathophysiology of PCOS.

## Introduction

Polycystic ovary syndrome (PCOS) is a complex endocrine disorder that affects approximately 10% of women during their reproductive age [1]. It is clinically characterized by a heterogeneous combination of symptoms, which notably include menstrual irregularities, hyperandrogenism, and the presence of polycystic ovarian morphology [2]. While traditionally recognized for its reproductive manifestations, PCOS significantly impacts metabolic health, leading to increased predisposition to insulin resistance, obesity, type 2 diabetes mellitus (T2DM), dyslipidemia, and heightened cardiovascular disease (CVD) risk [3].

Although the precise etiology of PCOS remains incompletely understood, evidence increasingly emphasizes its close relationship with metabolic dysfunction, particularly insulin resistance, obesity, and heightened susceptibility to T2DM [4]. Insulin resistance - a pathological condition defined by diminished tissue sensitivity to insulin, resulting in impaired glucose regulation [5] - is central to PCOS pathology, affecting up to 70% of women diagnosed with the syndrome [6]. The underlying mechanisms contributing to insulin resistance in PCOS are multifaceted, encompassing genetic predisposition, adiposity-driven inflammation, and altered secretion profiles of adipokines and cytokines [7,8].

Among the inflammatory mediators implicated in PCOS pathophysiology, interleukin-6 (IL-6), a multifunctional cytokine known for its regulatory roles in inflammation, immune modulation, and metabolic processes, has garnered significant research interest. Elevated IL-6 concentrations have been consistently linked with chronic inflammation and obesity, as well as metabolic disorders such as T2DM and CVD [9]. Consequently, IL-6 is increasingly recognized as a potential player in the pathogenesis of insulin resistance observed in PCOS.

Despite these promising insights, the specific role and clinical relevance of IL-6 within the context of PCOS remain insufficiently understood, particularly regarding comparative analyses of IL-6 levels in patients with differing insulin resistance statuses. This gap underscores the critical need for targeted investigations that can clarify the potential utility of IL-6 as a diagnostic or prognostic biomarker of insulin resistance severity in PCOS populations. This study aims to address this critical research void by systematically examining IL-6 concentrations among PCOS patients, differentiating between individuals with and without insulin resistance. Specifically, it seeks to explore the correlation of IL-6 levels with the severity of insulin resistance and to assess the cytokine's potential as a diagnostic biomarker within this patient group. Through this comprehensive analysis, the study aims to deepen the understanding of inflammatory mechanisms underlying insulin resistance in PCOS, thereby informing future therapeutic strategies and clinical interventions.

## Materials and methods

Study design

This was a cross-sectional study conducted at the Departments of Medicine and Gynecology, Mardan Medical Complex Teaching Hospital, Khyber Pakhtunkhwa, Pakistan, from February 2024 to January 2025. A convenience sampling method was utilized.

Inclusion criteria

Female patients aged between 18 and 45 years diagnosed with PCOS as per the Rotterdam criteria were included. These criteria necessitated at least two of the following conditions: oligo- or anovulation, clinical or biochemical hyperandrogenism, and polycystic ovarian morphology on ultrasound [10].

Exclusion criteria

Patients were excluded if they had concurrent endocrine disorders such as Cushing's syndrome, congenital adrenal hyperplasia, or thyroid dysfunction; were receiving medications affecting insulin sensitivity or IL-6 levels (e.g., glucocorticoids or insulin-sensitizing drugs); or were pregnant or breastfeeding.

Grouping of participants

Participants were divided into two groups based on the presence or absence of insulin resistance. Insulin resistance was assessed using the Homeostatic Model Assessment of Insulin Resistance (HOMA-IR), with a cutoff threshold of ≥2.5 to define insulin resistance [11,12].

Data collection

Demographic details, family history of diabetes and PCOS, body mass index (BMI), and clinical symptoms were collected using structured interviews and standardized questionnaires. Blood samples (five milliliters each of random and fasting venous blood) were collected from all participants.

Laboratory investigations

Serum IL-6 and insulin levels were measured via enzyme-linked immunosorbent assay (ELISA). Other biochemical analyses, including testosterone, luteinizing hormone (LH), follicle-stimulating hormone (FSH), prolactin, anti-Müllerian hormone (AMH), sex hormone-binding globulin (SHBG), glycated hemoglobin (HbA1c), fasting glucose, lipid profiles (total cholesterol, triglycerides, high-density lipoprotein (HDL), low-density lipoprotein (LDL)), C-reactive protein (CRP), serum creatinine, and blood urea, were conducted using Roche Cobas e411 and c111 analyzers (Roche Diagnostics, Basel, Switzerland). Hematological parameters, such as hemoglobin, platelet count, total leukocyte count, and differential leukocyte counts (neutrophils, lymphocytes, monocytes, eosinophils, basophils), were analyzed with a Sysmex XN-330 hematological analyzer (Sysmex, Kobe, Japan).

Biomarker calculations

The HOMA-IR was calculated using the formula: HOMA-IR = fasting insulin (μU/mL) × fasting glucose (mg/dL) ÷ 405 [13]. The total lipid profile was calculated as follows: total lipid profile = (2.27 × total cholesterol) + triglycerides + 62.3. Non-HDL cholesterol was derived by subtracting HDL from total cholesterol, while very-low-density lipoprotein (VLDL) cholesterol was calculated as triglycerides ÷ 5.

Statistical analysis

Continuous variables were presented as means and standard deviations, whereas categorical variables were summarized using frequencies and percentages. Differences between groups were analyzed using chi-square tests for categorical variables and independent sample t-tests or Mann-Whitney U tests for continuous variables based on the distribution of the data. Pearson correlation coefficients were used for correlation analyses. Simple linear regression analyses were performed for statistically significant correlations, followed by multiple linear regression analyses to adjust for confounding factors. Relationships between IL-6 levels and insulin resistance were explored using simple linear regression models visualized through scatter plots. Mean IL-6 levels and variability between groups were depicted graphically. Receiver operating characteristic (ROC) curve analysis was performed to assess the diagnostic performance of IL-6 in predicting insulin resistance, calculating the area under the curve (AUC). A p-value <0.05 was considered statistically significant. Statistical analyses were conducted using Statistical Product and Service Solutions (SPSS, version 29.0; IBM SPSS Statistics for Windows, Armonk, NY).

Ethical considerations

This study was carried out in accordance with ethical guidelines approved by the Institutional Review Board (IRB) NO467/BKMC. Informed consent was obtained from all participants prior to data collection, and confidentiality was ensured by anonymizing personal identifiers. No additional financial or medical burdens were placed on the participants, as the biomarker analysis was funded.

## Results

In this study of 112 PCOS patients, significant differences were observed between those with insulin resistance (44 patients, 39.29%) and those without (68 patients, 60.71%). Notably, within the insulin-resistant group, no individuals fell into the normal BMI range (18.5-24.9), a stark contrast to the 44.11% (30 patients) in the non-insulin-resistant group. Furthermore, 86.36% (38 patients) of the insulin-resistant group had a BMI of 30.0-39.9. Oily skin was reported in 70.45% (31 patients) of insulin-resistant patients versus 44.12% (30 patients) in the non-resistant group, skin tags in 75.00% (33 patients) versus 39.71% (27 patients), mood disturbances in 61.36% (27 patients) versus 36.76% (25 patients), and fatigue in 43.18% (19 patients) versus 19.11% (13 patients). Polycystic ovaries were significantly more prevalent in the insulin-resistant group at 77.27% (34 patients) compared to 45.58% (31 patients) in the non-resistant group, and the hirsutism score was slightly higher in the insulin-resistant group (12.55±5.81) compared to the non-resistant group (12.05±4.81). Table 1 presents the comparative characteristics of PCOS patients.

**Table 1:** Comparative Characteristics of PCOS Patients With and Without Insulin Resistance: Demographics, Clinical Features, and Insulin Sensitivity Outcomes Data are presented as frequency and percentage or as mean and standard deviation HOMA-IR: Homeostasis Model Assessment of Insulin Resistance; PCOS: Polycystic ovary syndrome P value <0.05 is statistically significant. P value with ** indicates statistical significance.

Characteristics	All Patients	Non-insulin Resistance HOMA-IR ≤2.5	Insulin Resistance HOMA-IR ≥2.5	Statistical Test	Test Statistics	P-value
Total Patients	112 (100)	68 (60.71)	44 (39.29)	Not applicable	Not applicable	Not applicable
Age (Years)	28.34±4.68	28.28±4.44	28.43±5.08	t	1.25	0.21
Body Mass Index (kg/m²)						
18.5-24.9	30 (26.78)	30 (44.11)	0 (0.00)	χ²	78.14	0.009**
25.0-29.9	44 (39.28)	38 (55.88)	6 (13.63)			
30.0-39.9	38 (33.92)	0 (0.00)	38 (86.36)			
Blood Pressure (mmHg)						
Diastolic Pressure	77.75±1.77	77.63±1.76	77.39±1.79	t	1.58	0.12
Systolic Pressure	115.44±2.88	115.25±2.93	115.73±2.78	t	1.26	0.21
Family History of PCOS	52 (46.42)	30 (44.11)	22 (50.00)	χ²	0.61	0.43
Symptoms						
Acne	60 (53.57)	36 (52.94)	24 (54.55)	χ²	2.53	0.11
Oily Skin	61 (54.46)	30 (44.12)	31 (70.45)	χ²	9.36	0.002**
Skin Tags	60 (53.57)	27 (39.71)	33 (75.00)	χ²	7.65	0.006**
Mood Disturbances	52 (46.42)	25 (36.76)	27 (61.36)	χ²	8.73	0.003**
Fatigue	32 (28.57)	13 (19.11)	19 (43.18)	χ²	6.62	0.01**
Pelvic Pain	22 (19.64)	13 (19.12)	9 (20.45)	χ²	2.58	0.11
Ovulation Problems	94 (83.93)	57 (83.82)	37 (84.09)	χ²	0.48	0.49
Irregular Menstrual Cycles	99 (88.39)	60 (88.24)	39 (88.64)	χ²	0.02	0.89
Polycystic Ovaries	65 (58.03)	31 (45.58)	34 (77.27)	χ²	5.31	0.021**
Hirsutism Score	12.34±5.06	12.05±4.81	12.55±5.81	t	2.01	0.049**

IL-6 levels were significantly higher in insulin-resistant patients (287.00±84.33 pg/mL) than in non-resistant patients (159.16±52.36 pg/mL). Differences in LH/FSH ratio, prolactin, anti-Müllerian hormone, and sex hormone-binding globulin highlighted worse profiles for insulin-resistant individuals. Total lipids, triglycerides, fasting blood glucose, and insulin levels further emphasized metabolic disparities, with insulin resistance linked to higher alkaline phosphatase and lower hemoglobin levels. Table 2 presents the biomarker profiles in PCOS patients.

**Table 2:** Biomarker Profiles in PCOS Patients: A Comparative Analysis of Insulin Sensitivity and Resistance Data are presented as mean and standard deviation. HOMA-IR: Homeostasis Model Assessment of Insulin Resistance; PCOS: Polycystic ovary syndrome P value <0.05 is statistically significant. P value with ** indicates statistical significance.

Biomarkers	All Patients	Non-insulin Resistance HOMA-IR ≤2.5	Insulin Resistance HOMA-IR ≥2.5	Statistical Test	Test Statistics	P-value
Interleukins-6 (pg/mL)	209.38±91.34	159.16±52.36	287.00±84.33	t	3.21	0.002**
C-Reactive Protein (mg/L)	1.94±0.59	2.02±0.62	1.82±0.54	t	1.36	0.18
Luteinizing Hormone (mIU/mL)	13.23±5.07	12.67±5.12	14.09±4.91	t	0.72	0.47
Follicle-Stimulating Hormone (mIU/mL)	5.43±1.49	5.38±1.51	5.52±1.48	t	1.48	0.14
LH/FSH Ratio	1.26±2.60	2.12±1.28	2.73±1.91	t	2.21	0.03**
Prolactin (ng/mL)	20.67±10.30	18.96±9.93	23.31±10.42	t	2.17	0.031**
Anti-Müllerian hormone (ng/mL)	3.67±1.41	3.54±1.34	3.85±4.51	t	2.05	0.04**
Sex Hormone-Binding Globulin (nmol/L)	41.22±14.45	48.10±14.39	30.59±5.10	t	4.92	0.00**
Total Testosterone (nmol/L)	0.66±0.26	0.64±0.25	0.65±0.28	t	0.91	0.37
Total Lipids (mg/dL)	665.20±46.80	653.21±45.61	683.09±42.11	t	3.37	0.001**
Cholesterol (mg/dL)	180.00±17.77	180.43±12.13	179.34±16.14	t	1.75	0.08
Triglycerides (mg/dL)	194.30±22.70	18.65±12.13	213.86±21.37	t	5.11	0.00**
High-Density Lipids (mg/dL)	42.80±3.05	42.93±3.12	42.61±2.98	t	0.44	0.66
Non-High Density Lipids (mg/dL)	137.20±17.71	137.50±18.82	136.73±16.03	t	1.98	0.05**
Low-Density Lipids (mg/dL)	94.63±17.57	86.78±11.99	106.77±18.01	t	2.59	0.01**
Very Low-Density Lipids (mg/dL)	38.86±4.54	36.32±2.42	42.77±4.27	t	5.23	0.00**
HBA1C (%)	5.22±0.19	5.21±0.18	5.22±0.21	t	0.76	0.45
Fasting Blood Glucose (mg/dL)	87.27±10.54	80.04±3.09	98.43±7.86	t	7.45	0.00**
Fasting Insulin (μU/mL)	14.18±6.17	9.47±1.58	21.45±2.29	t	6.28	0.01**
HOMA-IR	3.15±1.70	1.85±0.32	5.16±0.71	t	9.12	0.00**
Alanine Aminotransferase (U/L)	26.56±4.90	26.85±4.96	26.11±4.85	t	0.53	0.59
Alkaline Phosphatase (U/L)	107.43±36.56	85.09±15.33	141.95±32.78	t	8.17	0.00**
Serum Albumin (g/dL)	4.19±0.17	4.19±0.18	4.19±0.17	t	0.55	0.58
Total Bilirubin (mg/dL)	0.82±0.04	0.82±0.04	0.81±0.04	t	1.81	0.07
Serum Creatinine (mg/dL)	0.82±0.07	0.82±0.09	0.81±0.07	t	0.33	0.74
Blood Urea (mg/dL)	23.25±2.62	23.50±2.56	22.82±2.69	t	0.7	0.48
Hemoglobin (g/dL)	9.90±0.89	10.26±0.71	9.34±0.85	t	6.57	0.00**
Total Leukocyte Count (cells/μL)	8491±1043	8463±1024	8533±1083	t	0.63	0.53
Neutrophils (cells/μL)	5264±646	5247±634	5290±671	t	0.51	0.61
Lymphocytes (cells/μL)	2462±302	2454±296	2474±314	t	0.37	0.71
Monocytes (cells/μL)	424±52	423±51	426±54	t	0.69	0.49
Eosinophils (cells/μL)	169±20	169±20	169±21	t	0.71	0.48
Basophils (cells/μL)	169±20	169±21	170±21	t	0.63	0.53
Platelets (cells/μL)	270,009±51,146	273,098±47,517	265,234±56,535	t	0.77	0.44

IL-6 correlated strongly with BMI (correlation coefficient=0.65, p<0.001) and showed positive correlations with symptoms such as oily skin, skin tags, mood disturbances, and fatigue. Metabolic correlations with total lipids, triglycerides, low-density lipids, and fasting glucose and insulin suggested IL-6's role in insulin resistance. Negative correlations with hemoglobin levels and a positive association with polycystic ovaries indicated hormonal imbalances in PCOS. Table 3 presents the correlation analysis of IL-6 with PCOS indicators.

**Table 3:** Correlation Analysis of IL-6 With PCOS Indicators P value <0.05 is statistically significant. P value with ** indicates statistical significance. HOMA-IR: Homeostasis Model Assessment of Insulin Resistance; IL-6: Interleukin-6; PCOS: Polycystic ovary syndrome

Characteristics	Correlation Coefficient	P-value
Age (Years)	0.00	0.924
Body Mass Index (kg/m²)	0.65	0.00**
Diastolic Pressure (mmHg)	0.01	0.843
Systolic Pressure (mmHg)	0.09	0.341
Family History of PCOS	-0.03	0.723
Acne	-0.12	0.205
Oily Skin	0.29	0.001**
Skin Tags	0.33	0.041**
Mood Disturbances	0.36	0.03**
Fatigue	0.29	0.004**
Pelvic Pain	0.11	0.212
Ovulation Problems	0.21	0.11
Irregular Menstrual Cycles	0.13	0.41
Polycystic Ovaries	0.56	0.003**
Hirsutism Score	0.34	0.024**
Hemoglobin (g/dL)	-0.39	0.00**
Total Leukocyte Count (cells/μL)	-0.048	0.618
Neutrophils (cells/μL)	-0.048	0.619
Lymphocytes (cells/μL)	-0.047	0.62
Monocytes (cells/μL)	-0.048	0.616
Eosinophils (cells/μL)	-0.048	0.612
Basophils (cells/μL)	-0.048	0.612
Platelets (cells/μL)	-0.059	0.535
C-Reactive Protein (mg/L)	-0.14	0.137
Luteinizing Hormone (mIU/mL)	0.13	0.151
Follicle-Stimulating Hormone (mIU/mL)	0.05	0.572
LH/FSH Ratio	0.08	0.36
Prolactin (ng/mL)	0.47	0.004**
Anti-Müllerian Hormone (ng/mL)	0.03	0.715
Sex Hormone-Binding Globulin (nmol/L)	-0.36	0.00**
Total Testosterone (nmol/L)	0.04	0.619
Total Lipids (mg/dL)	0.28	0.002**
Cholesterol (mg/dL)	0.02	0.774
Triglycerides (mg/dL)	0.53	0.00**
High-Density Lipids (mg/dL)	-0.01	0.859
Non-High-Density Lipids (mg/dL)	0.03	0.75
Low-Density Lipids (mg/dL)	0.36	0.00**
Very Low-Density Lipids (mg/dL)	0.53	0.00**
HBA1C (%)	-0.03	0.754
Fasting Blood Glucose (mg/dL)	0.60	0.00**
Fasting Insulin (μU/mL)	0.66	0.00**
HOMA-IR	0.67	0.00**
Alanine Aminotransferase (U/L)	0.00	0.94
Alkaline Phosphatase (U/L)	0.53	0.00**
Serum Albumin (g/dL)	-0.00	0.966
Total Bilirubin (mg/dL)	-0.09	0.347
Serum Creatinine (mg/dL)	-0.08	0.371
Blood Urea (mg/dL)	-0.03	0.735

In the analysis of predictors for IL-6 levels through simple linear regression, significant associations were found with several variables. Among the variables, the BMI showed the strongest positive correlation (B=10.89, Beta=0.65, p<0.001), whereas in multiple linear regression, HOMA-IR (B=19.35, Beta=0.48, p=0.029) was identified as a significant predictor of IL-6 levels. Table 4 presents the simple and multi-linear regression analysis of predictors influencing IL-6 levels in PCOS patients.

**Table 4:** Simple and Multi-Linear Regression Analysis of Predictors Influencing IL-6 Levels in PCOS Patients HOMA-IR: Homeostasis Model Assessment of Insulin Resistance; IL-6: Interleukin-6; PCOS: Polycystic ovary syndrome

Model	Unstandardized Coefficients		Standardized Coefficients	95%CI		t-value	P-value
	B	Std. Error	Beta	Lower	Upper		
Simple Linear Regression Analysis							
Body Mass Index (kg/m²)	10.89	1.55	0.65	7.81	13.98	6.99	0.00
Polycystic Ovaries	18.21	4.91	0.56	12.31	19.44	3.46	0.003
Hirsutism Score	7.98	3.75	0.34	4.21	11.36	3.01	0.024
Oily Skin	9.09	3.46	0.29	5.28	13.61	5.55	0.001
Skin Tags	16.96	6.31	0.33	9.56	21.65	4.91	0.041
Mood Disturbance	6.17	2.22	0.36	2.29	9.91	3.76	0.03
Fatigue	3.39	1.44	0.29	1.96	5.36	1.84	0.004
Sex Hormone-Binding Globulin	-2.32	0.56	-0.36	-3.43	-1.21	-4.14	0.00
Prolactin (ng/mL)	2.40	0.81	0.47	0.79	4.01	2.95	0.004
Total Lipids (mg/dL)	0.55	0.17	0.28	0.20	0.90	3.11	0.002
Triglycerides (mg/dL)	2.16	0.32	0.53	1.52	2.80	6.69	0.00
Low-Density Lipids (mg/dL)	1.89	0.46	0.36	0.97	2.80	4.10	0.00
Very Low-Density Lipids (mg/dL)	10.82	1.61	0.53	7.62	14.03	6.69	0.00
Fasting Blood Glucose (mg/dL)	5.23	0.65	0.60	3.93	6.54	7.95	0.00
Fasting Insulin (μU/mL)	9.89	1.05	0.66	7.80	11.97	9.41	0.00
HOMA-IR	35.96	3.79	0.67	28.44	43.47	9.48	0.00
Alkaline Phosphatase (U/L)	1.32	0.20	0.53	0.92	1.72	6.56	0.00
Hemoglobin (g/dL)	-40.66	8.95	-0.39	-58.41	-22.92	-4.54	0.00
Multi-Linear Regression Analysis							
BMI (kg/m²)	8.61	2.09	0.37	6.42	13.78	4.41	0.043
Polycystic Ovaries	6.91	2.46	0.39	2.91	9.76	2.33	0.003
Prolactin (ng/mL)	1.65	0.74	0.26	0.16	3.13	2.20	0.041
HOMA-IR	19.35	4.11	0.48	13.91	24.96	5.99	0.029

**Figure 1:**
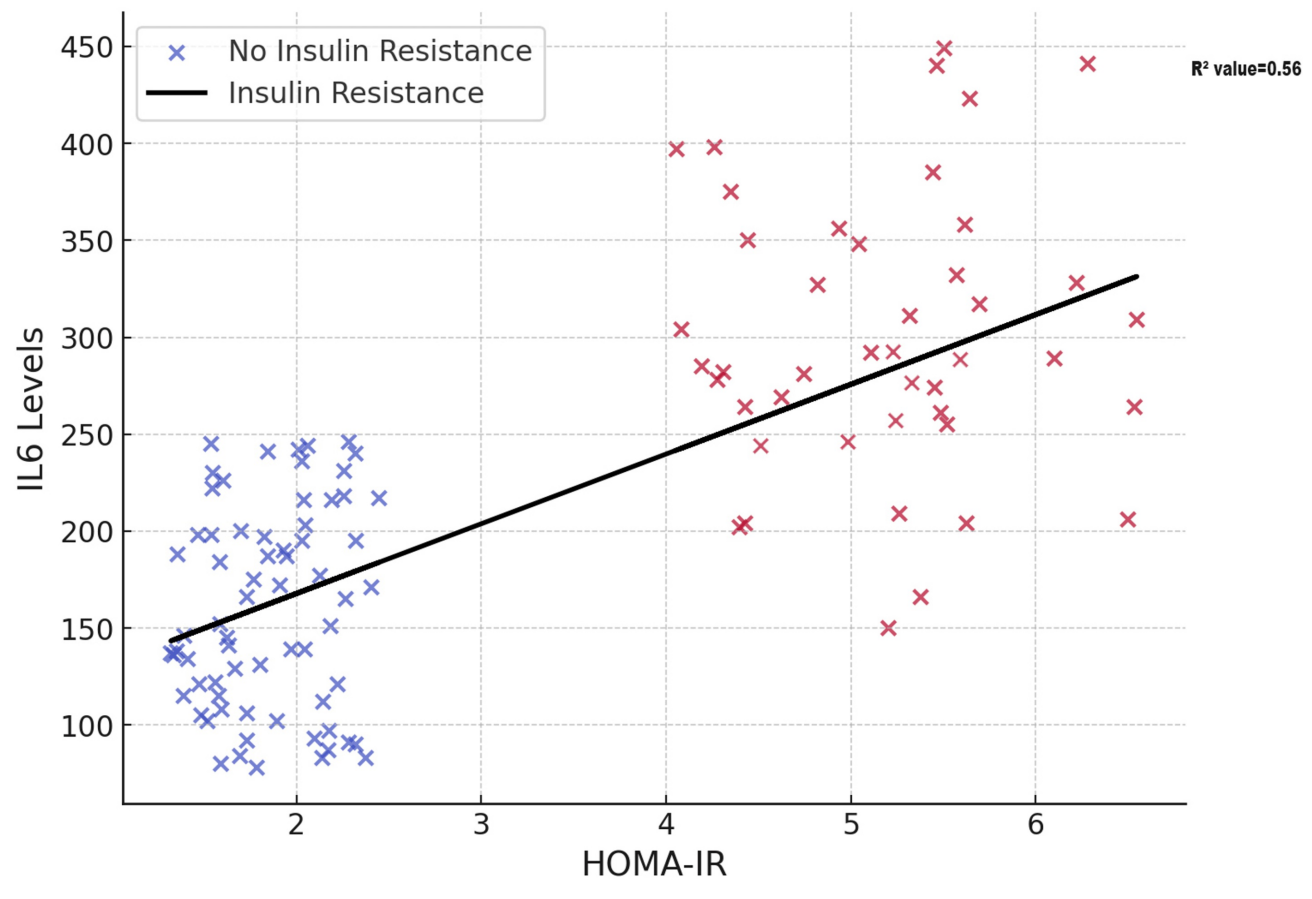
Relationship Between HOMA-IR and IL-6 Levels in PCOS Patients Simple scatter with fit line of HOMA-IR by IL-6 HOMA-IR: Homeostasis Model Assessment of Insulin Resistance; IL-6: Interleukin-6; PCOS: Polycystic ovary syndrome

Analysis of the relationship between IL-6 levels and insulin resistance revealed a moderate positive correlation, with a linear model (R²=0.56) explaining 56% of the variation in IL-6 levels as HOMA-IR increased. Figure 1 presents the relationship between HOMA-IR and IL-6 levels in PCOS patients.

**Figure 2:**
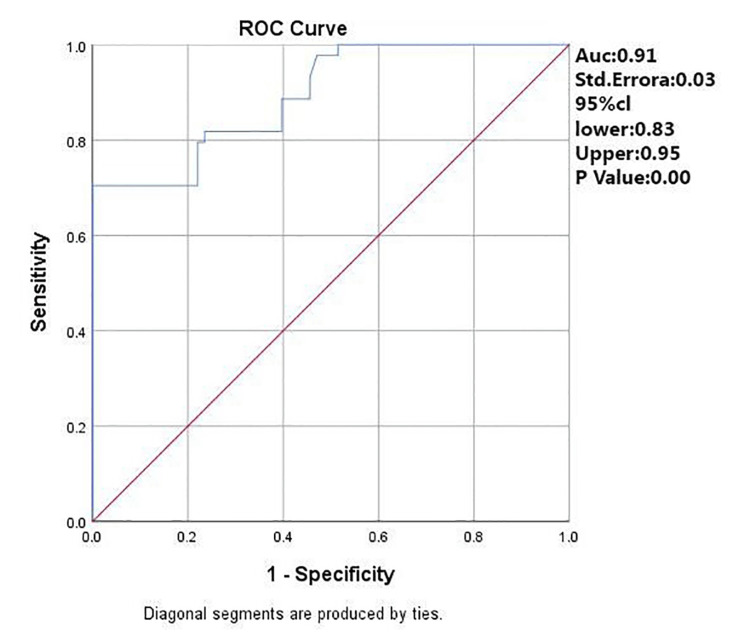
Evaluation of IL-6 as Diagnostic Test Performance for Insulin-Resistant Patients Using ROC Curve Analysis IL-6: Interleukin-6; ROC: Receiver operating characteristic

A ROC curve analysis revealed excellent diagnostic performance (AUC=0.91), suggesting the classifier's effectiveness in distinguishing between classes. Figure 2 presents the evaluation of IL-6 as a diagnostic test for insulin-resistant patients using ROC curve analysis.

**Figure 3:**
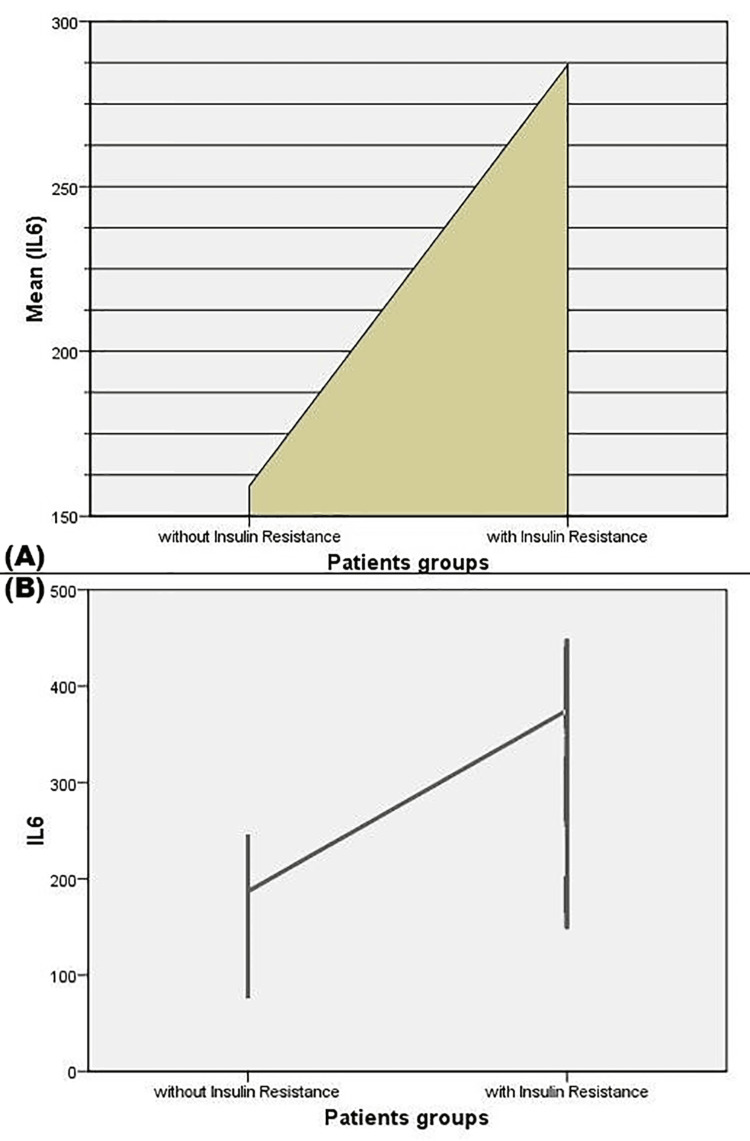
Comparison of Mean IL-6 levels Levels Between Patient Groups With and Without Insulin Resistance (A) Mean IL-6 levels in patients with and without insulin resistance; (B) IL-6 levels with error bars in patients with and without insulin resistance IL-6: Interleukin-6

Graphical analysis underscored significant differences in mean IL-6 levels between insulin-resistant and non-resistant patients, with notable variability in IL-6 levels among the insulin-resistant group, indicated by larger error bars. This comprehensive analysis highlights IL-6's significant association with PCOS symptoms, metabolic imbalances, and insulin resistance, underscoring its potential role in the pathophysiology of PCOS. Figure 3 presents the comparison of mean IL-6 levels between patient groups with and without insulin resistance.

## Discussion

The comparative analysis of IL-6 levels among patients diagnosed with PCOS represents a significant advancement in elucidating the complex interplay between inflammatory pathways and metabolic dysfunction characteristic of PCOS. The findings from this study, particularly the differentiation between PCOS patients with and without insulin resistance, underscore the critical role of IL-6 as an inflammatory mediator and highlight the multifactorial aspects inherent in the pathophysiology of PCOS.

The observation of elevated IL-6 levels in insulin-resistant PCOS patients reported in our study aligns closely with previous research by Escobar-Morreale et al., who similarly documented increased IL-6 concentrations among women with PCOS, attributing this elevation to heightened inflammatory responses driven by insulin resistance [14]. This alignment further supports the hypothesis that insulin resistance intensifies the inflammatory milieu in PCOS, exacerbating both metabolic disturbances and reproductive dysfunction. Additionally, our results corroborate the findings of González et al., who identified elevated IL-6 as a marker indicative of metabolic abnormalities among PCOS patients, reinforcing the association between heightened IL-6 levels and metabolic dysregulation within this patient population [15].

Our results demonstrating significantly elevated IL-6 levels in PCOS patients exhibiting insulin resistance complement the narrative established by Peng et al., which emphasized IL-6 as a critical mediator of inflammation in PCOS and delineated its correlation with the severity of insulin resistance and clinical manifestations [16]. Additionally, the positive correlation observed between IL-6 concentrations and BMI in our analysis is consistent with prior findings from Peng et al., who posited that increased adiposity significantly contributes to elevated inflammatory markers within the PCOS context [16]. Notably, the absence of individuals with normal BMI in our insulin-resistant subgroup further accentuates the complex interrelation between obesity, insulin resistance, and inflammation in PCOS.

Moreover, the relationship identified between elevated IL-6 concentrations and specific clinical manifestations of PCOS, such as oily skin and mood disturbances, aligns with observations by Aramesh et al., who noted mood disturbances in patients with PCOS [17]. Although Aramesh et al. did not evaluate IL-6 directly, our study extends their findings by suggesting that underlying inflammation potentially exacerbates the severity of such symptoms. Further support for IL-6's involvement in clinical manifestations and hormonal imbalance in PCOS can be derived from Al-Musawy et al., who detailed the disruptive influence of IL-6 on hormonal homeostasis within the PCOS patient population [18].

In addition, our findings of increased lipid profile parameters, fasting blood glucose, and insulin concentrations among insulin-resistant PCOS subjects are consistent with data reported by Dahan et al., who explored the interrelation between insulin resistance and hyperinsulinemia among women with PCOS. Their analysis concluded that PCOS patients exhibited significantly greater insulin resistance relative to ovulatory controls, even after adjusting for BMI, fasting glucose, and insulin concentrations [19]. Furthermore, Li et al. extensively characterized insulin resistance in PCOS, highlighting elevated fasting blood glucose, insulin concentrations, and increased HOMA-IR scores, alongside BMI, dyslipidemia, and hormonal disturbances [20]. These observations emphasize the extensive metabolic disturbances associated with insulin resistance in PCOS patients, reinforcing the importance of comprehensive metabolic assessments and cardiovascular risk evaluations as emphasized by recent guidelines [21]. Moreover, research by Stepto et al. further substantiates the deleterious impact of insulin resistance on metabolic health among PCOS patients, advocating for targeted interventions to address these metabolic aberrations and mitigate associated cardiovascular risks [22].

The intricate relationship between IL-6 levels and insulin resistance severity, as illustrated by scatter plot and ROC curve analyses in our study, suggests a complex, potentially nonlinear interaction that may hold significant clinical implications for the therapeutic management of insulin resistance among PCOS patients. Recognizing these nuanced interactions supports the rationale for adopting multifaceted therapeutic approaches targeting inflammation, metabolic dysfunction, and insulin resistance simultaneously in this population.

Despite the valuable insights provided, our study carries several limitations warranting acknowledgment. Firstly, the cross-sectional nature of this study design provides only a static evaluation at a single time point, limiting our ability to infer causation or track longitudinal changes. Future longitudinal studies would better clarify the temporal dynamics and causal roles of IL-6 in PCOS-associated insulin resistance. Secondly, our relatively small sample size, combined with the single-center recruitment strategy, constrains the generalizability and external validity of our findings. Thus, subsequent research incorporating larger, multicenter cohorts is recommended to validate and extend these results. Thirdly, the exclusion of participants with significant comorbidities restricts our capacity to evaluate how varying health conditions may independently affect IL-6 concentrations. Future studies should explicitly investigate IL-6 levels among PCOS patients with diverse comorbidities to better elucidate these associations. Lastly, a majority of the participants enrolled in our study had elevated BMI levels, introducing potential bias, as higher BMI independently contributes to elevated inflammatory markers, including IL-6, among PCOS patients. Consequently, the generalizability of our findings to populations with normal or lower BMI is limited. Subsequent research should aim to incorporate PCOS patients across broader BMI categories to achieve a more comprehensive understanding of IL-6 dynamics and clinical implications across different PCOS phenotypes.

## Conclusions

IL-6 plays a significant mediating role in the inflammatory pathways associated with PCOS, especially in patients with insulin resistance. This indicates that targeting IL-6 or its signaling pathways may offer a novel therapeutic approach for reducing inflammation and possibly improving insulin sensitivity in PCOS patients. The study concluded that PCOS patients with insulin resistance exhibited significantly higher levels of IL-6 compared to those without insulin resistance, suggesting a strong correlation between IL-6 levels and insulin resistance in the context of PCOS. This finding indicates that IL-6 may play a crucial role in exacerbating insulin resistance among PCOS patients, potentially contributing to the severity of PCOS symptoms.
